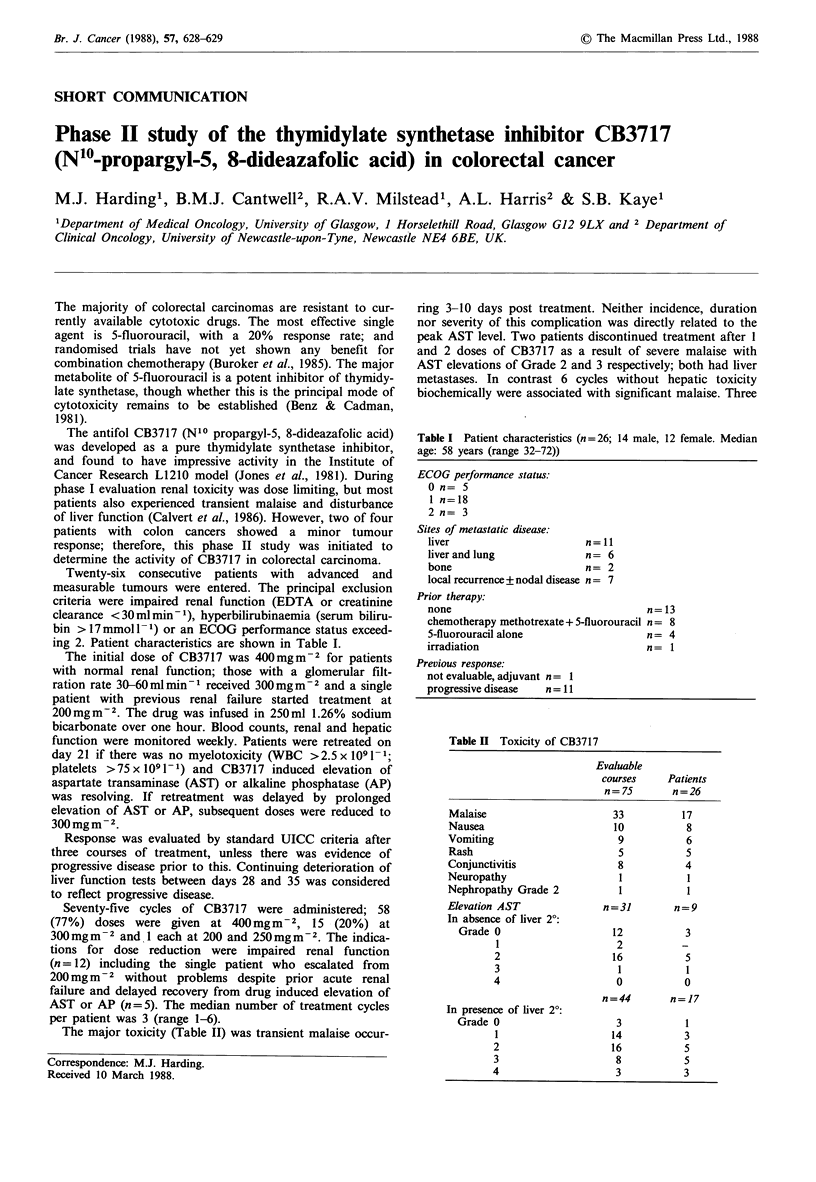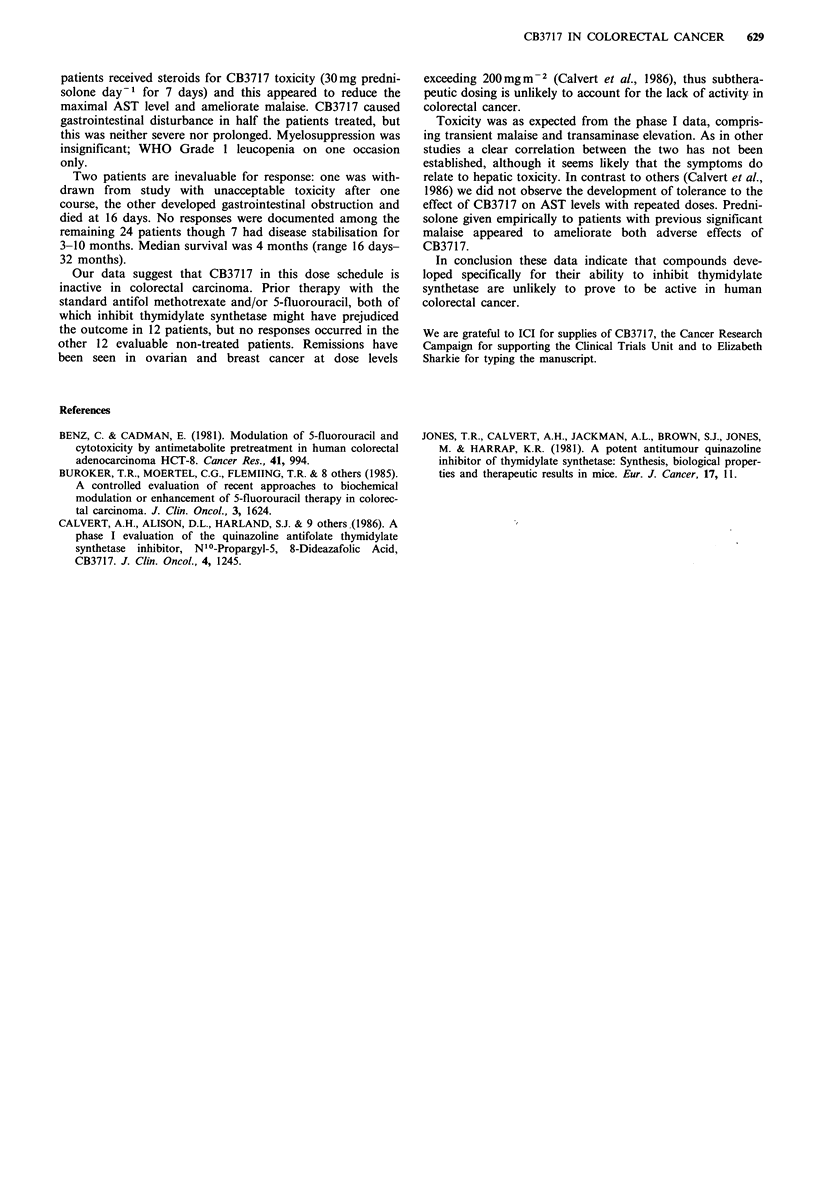# Phase II study of the thymidylate synthetase inhibitor CB3717 (N10-propargyl-5,8-dideazafolic acid) in colorectal cancer.

**DOI:** 10.1038/bjc.1988.143

**Published:** 1988-06

**Authors:** M. J. Harding, B. M. Cantwell, R. A. Milstead, A. L. Harris, S. B. Kaye

**Affiliations:** Department of Medical Oncology, University of Glasgow, UK.


Br. J. Cancer (1988), 57, 628–629 © The Macmillan Press Ltd., 1988

SHORT COMMUNICATION

### Phase II study of the thymidylate synthetase inhibitor CB3717 ($N^{10}$-propargyl-5, 8-dideazafolic acid) in colorectal cancer

M.J. Harding[1], B.M.J. Cantwell[2], R.A.V. Milstead[1], A.L. Harris[2] & S.B. Kaye[1]

[1]*Department of Medical Oncology, University of Glasgow, 1 Horselethill Road, Glasgow G12 9LX and* [2] *Department of Clinical Oncology, University of Newcastle-upon-Tyne, Newcastle NE4 6BE, UK.*

The majority of colorectal carcinomas are resistant to currently available cytotoxic drugs. The most effective single agent is 5-fluorouracil, with a 20% response rate; and randomised trials have not yet shown any benefit for combination chemotherapy (Buroker *et al.*, 1985). The major metabolite of 5-fluorouracil is a potent inhibitor of thymidylate synthetase, though whether this is the principal mode of cytotoxicity remains to be established (Benz & Cadman, 1981).

The antifol CB3717 ($N^{10}$ propargyl-5, 8-dideazafolic acid) was developed as a pure thymidylate synthetase inhibitor, and found to have impressive activity in the Institute of Cancer Research L1210 model (Jones *et al.*, 1981). During phase I evaluation renal toxicity was dose limiting, but most patients also experienced transient malaise and disturbance of liver function (Calvert *et al.*, 1986). However, two of four patients with colon cancers showed a minor tumour response; therefore, this phase II study was initiated to determine the activity of CB3717 in colorectal carcinoma.

Twenty-six consecutive patients with advanced and measurable tumours were entered. The principal exclusion criteria were impaired renal function (EDTA or creatinine clearance $<30\,ml\,min^{-1}$), hyperbilirubinaemia (serum bilirubin $>17\,mmol\,l^{-1}$) or an ECOG performance status exceeding 2. Patient characteristics are shown in Table I.

The initial dose of CB3717 was $400\,mg\,m^{-2}$ for patients with normal renal function; those with a glomerular filtration rate $30–60\,ml\,min^{-1}$ received $300\,mg\,m^{-2}$ and a single patient with previous renal failure started treatment at $200\,mg\,m^{-2}$. The drug was infused in 250 ml 1.26% sodium bicarbonate over one hour. Blood counts, renal and hepatic function were monitored weekly. Patients were retreated on day 21 if there was no myelotoxicity (WBC $>2.5\times10^9\,l^{-1}$; platelets $>75\times10^9\,l^{-1}$) and CB3717 induced elevation of aspartate transaminase (AST) or alkaline phosphatase (AP) was resolving. If retreatment was delayed by prolonged elevation of AST or AP, subsequent doses were reduced to $300\,mg\,m^{-2}$.

Response was evaluated by standard UICC criteria after three courses of treatment, unless there was evidence of progressive disease prior to this. Continuing deterioration of liver function tests between days 28 and 35 was considered to reflect progressive disease.

Seventy-five cycles of CB3717 were administered; 58 (77%) doses were given at $400\,mg\,m^{-2}$, 15 (20%) at $300\,mg\,m^{-2}$ and 1 each at 200 and $250\,mg\,m^{-2}$. The indications for dose reduction were impaired renal function ($n=12$) including the single patient who escalated from $200\,mg\,m^{-2}$ without problems despite prior acute renal failure and delayed recovery from drug induced elevation of AST or AP ($n=5$). The median number of treatment cycles per patient was 3 (range 1–6).

The major toxicity (Table II) was transient malaise occurring 3–10 days post treatment. Neither incidence, duration nor severity of this complication was directly related to the peak AST level. Two patients discontinued treatment after 1 and 2 doses of CB3717 as a result of severe malaise with AST elevations of Grade 2 and 3 respectively; both had liver metastases. In contrast 6 cycles without hepatic toxicity biochemically were associated with significant malaise. Three

**Table I** Patient characteristics ($n=26$; 14 male, 12 female. Median age: 58 years (range 32–72))

| | |
|---|---|
| *ECOG performance status:* | |
| 0 | $n=5$ |
| 1 | $n=18$ |
| 2 | $n=3$ |
| *Sites of metastatic disease:* | |
| liver | $n=11$ |
| liver and lung | $n=6$ |
| bone | $n=2$ |
| local recurrence ± nodal disease | $n=7$ |
| *Prior therapy:* | |
| none | $n=13$ |
| chemotherapy methotrexate + 5-fluorouracil | $n=8$ |
| 5-fluorouracil alone | $n=4$ |
| irradiation | $n=1$ |
| *Previous response:* | |
| not evaluable, adjuvant | $n=1$ |
| progressive disease | $n=11$ |

**Table II** Toxicity of CB3717

| | *Evaluable courses* $n=75$ | *Patients* $n=26$ |
|---|---|---|
| Malaise | 33 | 17 |
| Nausea | 10 | 8 |
| Vomiting | 9 | 6 |
| Rash | 5 | 5 |
| Conjunctivitis | 8 | 4 |
| Neuropathy | 1 | 1 |
| Nephropathy Grade 2 | 1 | 1 |
| *Elevation AST* | $n=31$ | $n=9$ |
| In absence of liver 2°: | | |
| Grade 0 | 12 | 3 |
| 1 | 2 | – |
| 2 | 16 | 5 |
| 3 | 1 | 1 |
| 4 | 0 | 0 |
| | $n=44$ | $n=17$ |
| In presence of liver 2°: | | |
| Grade 0 | 3 | 1 |
| 1 | 14 | 3 |
| 2 | 16 | 5 |
| 3 | 8 | 5 |
| 4 | 3 | 3 |

Correspondence: M.J. Harding.
Received 10 March 1988.

patients received steroids for CB3717 toxicity (30 mg prednisolone day$^{-1}$ for 7 days) and this appeared to reduce the maximal AST level and ameliorate malaise. CB3717 caused gastrointestinal disturbance in half the patients treated, but this was neither severe nor prolonged. Myelosuppression was insignificant; WHO Grade 1 leucopenia on one occasion only.

Two patients are inevaluable for response: one was withdrawn from study with unacceptable toxicity after one course, the other developed gastrointestinal obstruction and died at 16 days. No responses were documented among the remaining 24 patients though 7 had disease stabilisation for 3–10 months. Median survival was 4 months (range 16 days–32 months).

Our data suggest that CB3717 in this dose schedule is inactive in colorectal carcinoma. Prior therapy with the standard antifol methotrexate and/or 5-fluorouracil, both of which inhibit thymidylate synthetase might have prejudiced the outcome in 12 patients, but no responses occurred in the other 12 evaluable non-treated patients. Remissions have been seen in ovarian and breast cancer at dose levels exceeding 200 mg m$^{-2}$ (Calvert *et al.*, 1986), thus subtherapeutic dosing is unlikely to account for the lack of activity in colorectal cancer.

Toxicity was as expected from the phase I data, comprising transient malaise and transaminase elevation. As in other studies a clear correlation between the two has not been established, although it seems likely that the symptoms do relate to hepatic toxicity. In contrast to others (Calvert *et al.*, 1986) we did not observe the development of tolerance to the effect of CB3717 on AST levels with repeated doses. Prednisolone given empirically to patients with previous significant malaise appeared to ameliorate both adverse effects of CB3717.

In conclusion these data indicate that compounds developed specifically for their ability to inhibit thymidylate synthetase are unlikely to prove to be active in human colorectal cancer.

We are grateful to ICI for supplies of CB3717, the Cancer Research Campaign for supporting the Clinical Trials Unit and to Elizabeth Sharkie for typing the manuscript.

### References

BENZ, C. & CADMAN, E. (1981). Modulation of 5-fluorouracil and cytotoxicity by antimetabolite pretreatment in human colorectal adenocarcinoma HCT-8. *Cancer Res.*, **41**, 994.

BUROKER, T.R., MOERTEL, C.G., FLEMIING, T.R. & 8 others (1985). A controlled evaluation of recent approaches to biochemical modulation or enhancement of 5-fluorouracil therapy in colorectal carcinoma. *J. Clin. Oncol.*, **3**, 1624.

CALVERT, A.H., ALISON, D.L., HARLAND, S.J. & 9 others (1986). A phase I evaluation of the quinazoline antifolate thymidylate synthetase inhibitor, $N^{10}$-Propargyl-5, 8-Dideazafolic Acid, CB3717. *J. Clin. Oncol.*, **4**, 1245.

JONES, T.R., CALVERT, A.H., JACKMAN, A.L., BROWN, S.J., JONES, M. & HARRAP, K.R. (1981). A potent antitumour quinazoline inhibitor of thymidylate synthetase: Synthesis, biological properties and therapeutic results in mice. *Eur. J. Cancer*, **17**, 11.